# Induced Pluripotent Stem Cells and Genome-Editing Tools in Determining Gene Function and Therapy for Inherited Retinal Disorders

**DOI:** 10.3390/ijms232315276

**Published:** 2022-12-03

**Authors:** Daniela Benati, Amy Leung, Pedro Perdigao, Vasileios Toulis, Jacqueline van der Spuy, Alessandra Recchia

**Affiliations:** 1 Centre for Regenerative Medicine, Department of Life Sciences, University of Modena and Reggio Emilia, 41125 Modena, Italy; 2 UCL Institute of Ophthalmology, London EC1V 9EL, UK; 3 Center for Neuroscience and Cell Biology, University of Coimbra, 3004-504 Coimbra, Portugal

**Keywords:** retinal disorders, induced pluripotent stem cells, CRISPR/Cas

## Abstract

Inherited retinal disorders (IRDs) affect millions of people worldwide and are a major cause of irreversible blindness. Therapies based on drugs, gene augmentation or transplantation approaches have been widely investigated and proposed. Among gene therapies for retinal degenerative diseases, the fast-evolving genome-editing CRISPR/Cas technology has emerged as a new potential treatment. The CRISPR/Cas system has been developed as a powerful genome-editing tool in ophthalmic studies and has been applied not only to gain proof of principle for gene therapies in vivo, but has also been extensively used in basic research to model diseases-in-a-dish. Indeed, the CRISPR/Cas technology has been exploited to genetically modify human induced pluripotent stem cells (iPSCs) to model retinal disorders in vitro, to test in vitro drugs and therapies and to provide a cell source for autologous transplantation. In this review, we will focus on the technological advances in iPSC-based cellular reprogramming and gene editing technologies to create human in vitro models that accurately recapitulate IRD mechanisms towards the development of treatments for retinal degenerative diseases.

## 1. Introduction

For many years, animal models have been used in order to study the genotype–phenotype correlation in many genetic diseases, such as inherited retinal disorders (IRDs). However, animal models do not always reflect human disorders accurately and may have contributed to disappointing outcomes in clinical trials of drugs and/or therapies. As a valuable alternative to in vivo models, stem cell research has experienced rapid development in recent years. Embryonic stem cell-derived retinal pigment epithelium (RPE) and photoreceptors have been used to replace degenerated epithelia or cells in the eye. In several phase 1/2 clinical trials, RPE cells or photoreceptors have been administered to the patient eye by direct intravitreal or subretinal injection or embedded in a substrate for implantation [1].

Since large amounts of embryonic tissue are required to isolate stem cells, there has been an increased interest in the development of technologies to isolate different adult somatic cell candidates to be induced to pluripotent stem cells and further differentiated into retinal cells. The discovery of somatic cellular reprogramming into induced pluripotent stem cells (iPSC) [2,3] marked a milestone for the in vitro production of human tissue that has enabled the generation of patient-derived disease models [4,5,6,7]. Patients’ cells can be collected from different sources, reprogrammed to become pluripotent, and then differentiated into retinal cells to model in vitro IRDs.

In the past, to identify and characterize disease-specific phenotypes, iPSCs carrying genetic mutations were compared to wild-type iPSC lines. However, iPSCs derived from healthy donors or age-matched, sex-matched, or sibling donors do not faithfully reflect patients’ iPSCs. Indeed, past studies based on iPSC models unveiled major phenotypic variabilities derived from inter-individual comparison that confounds the investigation of such mechanisms and the evaluation of future therapeutics [6]. To date, the genome of patient-derived iPSC can be modified quite easily by the CRISPR/Cas9 system to establish a genetically defined condition, overcoming the genetic background variations between patient and control iPSCs. Thereby, isogenic iPSCs are strongly preferred to evaluate the genotype-to-phenotype correlation and can be used to test gene therapies and drugs for a potential treatment of the disease.

Here, we will review the technological advances in iPSC-based cellular reprogramming and gene editing technologies to create human in vitro models that accurately recapitulate IRD mechanisms towards the development of advanced therapy medical products (ATMP).

## 2. Reprogramming Induced Pluripotent Stem Cells

Since the original discovery of cellular reprogramming of somatic cells to pluripotency [2,8], iPSC technology has rapidly advanced to refine the process of human iPSC generation and differentiation to lineages of interest, including retinal cells. This has encompassed the development of alternate techniques for iPSC generation and the characterization of iPSCs derived from different cellular lineages with respect to their pluripotency and differentiation capabilities, all of which has a bearing on the potential clinical applications of iPSC technology, as well as the use of iPSCs as a platform to study gene function, hereditary disease and cell differentiation in vitro.

While iPSC generation was first achieved with the 4 canonical “Yamanaka factors” (OCT4, NANOG, KLF4, C-MYC) [8], downregulating p53 expression [9], promoting the expression of the mir302-367 cluster [10,11] and replacing C-MYC with non-transforming L-MYC factor [12] has advanced the efficiency of iPSC generation.

**Figure 1:**
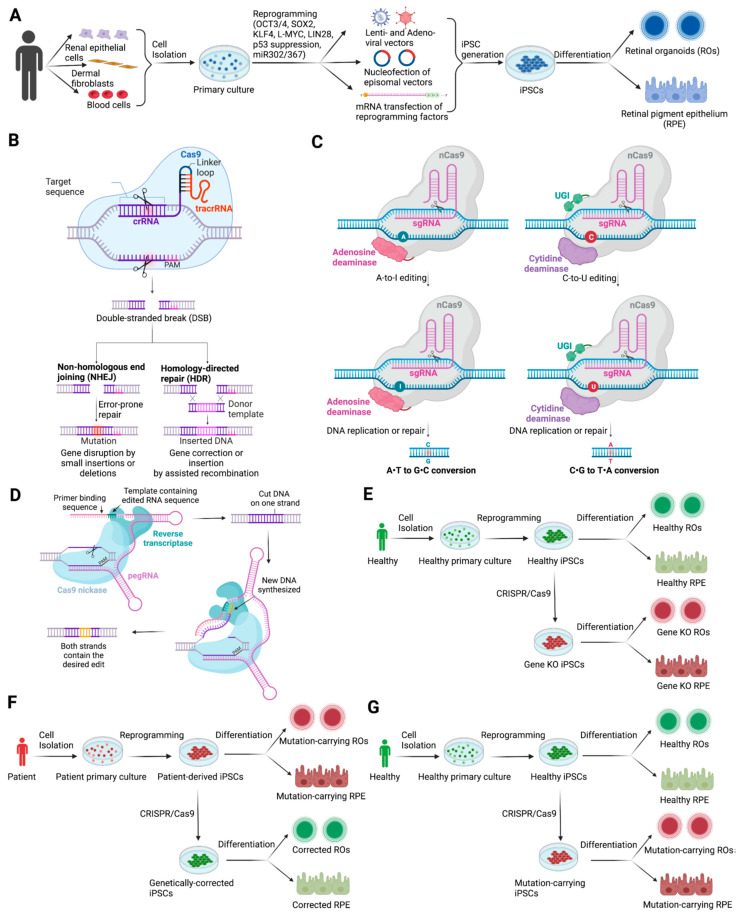
Generation of in vitro models to study inherited retinal dystrophies (IRDs). (**A**) Schematic diagram of sources of patient somatic cells, reprogramming methods into induced pluripotent stem cells (iPSCs), delivery methods of reprogramming factors and differentiation approaches for generating retinal organoids (ROs) and retinal pigment epithelium (RPE). (**B**) Introduction of DNA double-stranded breaks (DSB) by Cas9 endonuclease directed to a predetermined genomic locus by a small guide RNA (sgRNA) complementary to the target sequence. DSBs are resolved by either the non-homologous end joining (NHEJ) pathway or the homology-directed repair (HDR) pathway, which allows precise and scarless genetic modification. (**C**) CRISPR base editors. An inactive Cas9 nickase (nCas9) is fused to a deoxynucleotide deaminase that can precisely mediate nucleotide transitions without requiring the induction of DSBs or homology repair pathways. Adenosine base editors (ABE) and cytosine base editors (CBE) convert A-to-G or C-to-T, respectively, and thus promote targeted point mutations. (**D**) CRISPR prime editors. The Cas9 nickase is fused to a reverse transcriptase (RT), which directly transcribes a customized sequence into the genomic locus of interest. The desired template is encoded in a 3′-extension of the guide RNA [prime editing gRNA (pegRNA)], which primes with the target region and directs the RT-mediated synthesis of the newly edited DNA. (**E**–**G**) Strategies for generating iPSC isogenic pairs, using gene-editing technologies, aimed to: study the function of disease-causing mutations in IRDs by knocking out (KO) the normal wild-type (WT) gene in healthy iPSCs (**E**); correct of a disease-causing mutation in patient-derived iPSCs (**F**); insert of disease-causing mutations in healthy iPSCs (**G**).

In the past, iPSC generation methods involved the use of Lentiviral [8,13], Adenoviral [14] and Sendai viral vectors [15]. However, the delivery of reprogramming factors to target cells by simple nucleofection of plasmids or mRNA transiently expressing the reprogramming factors has significantly improved the genomic safety profile, viability and pluripotency of iPSCs [16,17,18] (Figure 1A).

### Source of Patient Cells for iPSC Reprogramming

Reprogramming of human somatic cells was originally achieved using human dermal fibroblasts [2]. Although skin fibroblasts remain a commonly-used source of patient samples for iPSC generation, successful reprogramming has been carried out with a vast array of different somatic cell types, including from more accessible tissues such as blood [19,20] and renal epithelial cells [21,22]. The latter is a particularly non-invasive technique for harvesting cells from patients (an especial consideration for paediatric patients), and as reprogramming techniques have advanced, it is possible to reliably establish sufficient numbers of iPSC clones from the relatively low numbers of renal epithelial cells present in urine samples, using episomal vectors [21].

The source of somatic cells for reprogramming appears to have no impact on the neuroretinal potential of the iPSC generated. Indeed, Capowski and colleagues investigated the retinal potential of nine blood and three fibroblast-derived iPSC lines and found that all iPSC lines were able to give rise to similar neuroretinal cultures [23]. Moreover, iPSC lines either from healthy donors or patients with various types of hereditary retinal disease have been derived from skin, blood and renal epithelial cells through different techniques and differentiated towards retinal lineages [24], as demonstrated by the formation of multi-layered retinal organoids (ROs) [25], RPE [26,27] and photoreceptors [28]. In addition to this, retinal development and retinopathies have also been successfully studied by utilizing iPSC from diverse cell sources such as conjunctival [27], dental pulp [29] and post-mortem adult Müller glia [30] cell sources (Table 1).

The accessibility of CRISPR/Cas technology to edit the genome of different species and organisms has been exploited to genetically modify the human iPSCs to model retinal disorders and to test in vitro drugs and therapies.

## 3. CRISPR/Cas9 Toolbox for iPSC Editing

### 3.1. CRISPR/Cas9 Editing System

The genome-editing systems are based on site-specific nucleases (SSN) programmed to engage a predetermined genomic locus and trigger a DNA double-strand break (DSB) [31]. Early efforts to genetically modify iPSC relied mostly on zinc-finger nucleases (ZFN) [32,33,34,35] or transcription activator-like effector nucleases (TALENs) [36,37,38,39]. Target DNA recognition by these SSN is mediated by the amino acid side chains of the zinc-fingers or TALE domains, which act as heterodimers fused to *FokI* nuclease to induce DSBs [31]. While innovative, these methods required the engineering of two SSN proteins to target a single site and thus were costly, time consuming and quite often inefficient.

In 2020, Jennifer Doudna and Emmanuelle Charpentier shared the Nobel Prize award for developing the precise CRISPR genome-editing technology. A great contribution to the characterization of the CRISPR system was carried out by Francisco Mojica and co-workers, who in 2005 reported the discovery of sequence similarity between the spacer regions of CRISPR and sequences of bacteriophages, archaeal viruses, and plasmids, shedding light on the function of CRISPR as an immune system [40]. The CRISPR—Clustered Regularly Interspaced Short Palindromic Repeats—endonuclease system has become the gold standard method for genome-editing applications. In this system, gene editing is mediated by a CRISPR-associated (Cas) endonuclease directed to a predetermined genomic locus by a short guide RNA (gRNA), complementary to the target sequence (protospacer) [41]. The simplicity and low-cost of gRNA design significantly expedited the generation of gene editing tools, which is reflected in the exponential growth of CRISPR/Cas genome engineering applications ever since [42]. CRISPR/Cas activity requires the presence of a short sequence named the protospacer-adjacent motif (PAM), which differs according to the Cas subtype employed, just downstream of the protospacer. CRISPR mechanisms have naturally evolved across different bacterial strains, with over 33 different CRISPR family subtypes identified so far [43]. These are divided into two classes, in which class 2 CRISPR systems are distinguished by a single effector for target DNA engagement and cleavage activity. Within this family, *Streptococcus pyogenes* Cas9 (SpCas9) was the first endonuclease studied in its ability to perform targeted gene editing [44]. Today, SpCas9 remains the most efficient gene-editing tool [42] and the most widely employed in genetic engineering applications in iPSC [45].

Upon recognition of the target site, the Cas9 endonuclease domains HNH and RuvC nick each DNA strand and consequently trigger a DSB [41]. In most events, DSBs are resolved by the activation of the non-homologous end joining (NHEJ) pathway [46]. Here, nucleotide indels occur during the repair, which leads to a small modification of the target sequence. More rarely, a single DSB can result in the deletion of large regions positioned between micro-homologous sites by triggering the microhomology-mediated end joining (MMEJ) pathway. Initially established as a random process, studies have shown that NHEJ and MMEJ repair outcomes are highly reproducible, greatly depend on the target DNA sequence [47,48] and can even be predicted by deep learning algorithms [49,50,51]. When DNA breaks occur in a gene coding region, activation of NHEJ/MMEJ pathways results in a coding frameshift and consequent emergence of premature stop codons, which results in permanent gene disruption [52].

In contrast to these pathways, activation of the homology-directed repair (HDR) pathway, which allows precise and scarless genetic modification, is triggered by the co-delivery of a donor template with the intended mutation flanked by sequences homologous to the region spanning the DNA break [46] (Figure 1B). As HDR activation is mostly restricted to S/G2 stages of the replication cycle [53], this approach has only been effective on actively dividing cells, which naturally extends to iPSC lines.

However, high levels of HDR-mediated precise gene editing in iPSC have still proven to be a difficult task [54,55]. Critical settings identified over the past years for maximizing HDR efficiency have become valuable guidelines for designing-gene editing experiments in iPSCs. One is to trigger endonuclease cuts as close as possible—ideally within 10 bp—to the target region to ensure the knock-in of the intended gene modification [56]. This can, however, limit the application of CRISPR/Cas systems to regions with a PAM motif close enough to the targeted variant. Novel Cas effectors belonging to type V and VI of Class 2 CRISPR/Cas and SpCas9 variants with PAM recognition to non-NGG motifs have been isolated and engineered to expand the targeting regions of this gene-editing system [57,58,59,60]. In addition, to minimizing the cut-to-mutation distance, Paquet and colleagues also showed that HDR levels significantly increase when silent mutations are introduced in the donor template, either in the PAM motif or in the protospacer sequence, to avoid Cas9 recognition and re-cutting of the template following recombination [56].

The nature of the homologous donor template also seems to play a key role in successfully promoting HDR in iPSC. Double-stranded plasmid constructs or even bacterial artificial chromosomes with long homology arms (from 10 to 100 Kb) were primarily used to promote HDR in iPSC but with little efficiency, typically requiring the co-integration of selection cassettes such as fluorescent reporters or antibiotic resistance genes to enrich gene-edited populations [61]. The Cre/loxP recombinase [62,63] or Piggy bac transposase [64,65] systems have been harnessed to excise selection cassettes markers following cell selection but require a second intervention, making the procedure more time-consuming. In contrast, single-stranded templates appear to be optimal substrates to stimulate homologous recombination without the requirement for selection markers. Single-stranded deoxyoligonucleotide (ssODN) templates with short homology arms of 35–150 bp promote scarless knock-in in iPSC lines and have been widely used to generate gene-corrected isogenic iPSC with small gene modifications (<50 bp) [56]. HDR appears to be stimulated even further when ssODN templates are designed with asymmetric homology arms [66]. To support the editing tools, small-molecule drugs have also proven beneficial in improving gene-targeted knock-in in iPSC by modulating the cell cycle or interfering with DNA repair pathways to shift the balance towards HDR [67].

A major concern for the application of the CRISPR/Cas system is the potential off-target activity of the Cas9 endonuclease at unintended genomic loci [68], which could potentially exert genotoxic and cytotoxic effects. It is now generally established that direct delivery of Cas9 ribonucleoparticle (RNP) is the most suitable route for safe editing, greatly minimizing off-target occurrences by shortening the endonuclease activity window and avoiding long-term cell exposure to the gene-editing enzyme [69]. Still, detection of off-target modifications is presently a major requirement for the characterization of gene-edited cells. Most studies predicted the possible off-target events in silico and evaluated the risk of editing by Sanger sequencing [41,70]. However, whole genome sequencing is the best approach to fully elucidate the existence of off-target mutations [71] but usually discarded owing its high cost and time-consuming analysis.

So far, no study has reported the interference of unintended CRISPR/Cas9-mediated off-targets on the recapitulation of disease mechanisms in isogenic iPSC lines.

### 3.2. Next-Generation Editing Tools: CRISPR Base and Prime Editors

The versatility of the CRISPR/Cas9 system has enabled the bioengineering of next-generation editing tools that eliminate current limitations of the system.

David Liu and co-workers developed, in the last six years, two CRISPR-based systems to promote base transitions without requiring the induction of DSBs or homology repair pathways [72]. Formed by the fusion of an inactive Cas9 nickase—in which the RuvC nuclease domain is inactivated—to a deoxynucleotide deaminase, these so-called adenosine base editors (ABE) and cytosine base editors (CBE) can respectively convert A-to-G or C-to-T [73,74] (Figure 1C). Their outstanding ability to promote targeted point mutations greatly surpassed conventional HDR-mediated gene-editing methods [72] and are currently one of the most promising gene editing tools to tackle IRD mutations.

One major constraint of CRISPR base editors, however, is the requirement for a proximal PAM to position the targeted nucleotide in the ABE/CBE optimal editing window, ideally in positions 4–8 of the gRNA protospacer sequence for base editors that incorporate the SpCas9 nickase framework [72]. In addition, the sequence context might frequently not be favorable for scarless gene correction as bystander mutations can occur when neighboring A or C nucleotides positioned within the editing window are also targeted. Both limitations have been respectively improved with the formation of base editors with flexible editing windows or PAM requirements [75]. Together, ABE and CBE can target approximately 61% of known variants associated with human disease [72]. While novel base editors for induction of base transversions are in development, such as C-to-G base editors [76], nucleotide insertion/deletion have required a further expansion of the CRISPR toolbox, which recently included the prime editor (PE). Indeed, taking advantage of the unique ability of reverse transcriptase (RT) to synthesize DNA from RNA templates, David Liu and his team fused Cas9 nickase to murine leukaemia virus (MLV)-RT. This complex acts by directly copying a customized prime editing gRNA (pegRNA) into the genomic locus of interest [77] (Figure 1D). Prime editors can precisely install all types of point mutations, as well as insertions and deletions within a restricted length, and without bystander effects typically observed with base editors. Moreover, mutations can be installed at regions > 30 bp from the targeted sequence, which offers greater range and less dependence on PAM availability compared to endonuclease-mediated HDR or base editing [75].

### 3.3. Delivery of CRISPR/Cas9 Components to iPSC

CRISPR/Cas gene editing components can be delivered to cells by viral transduction or by chemical- or electroporation-based transfection. Nucleofection seems to be the most effective method to transfect primary cells or iPSC with Cas9 and gRNA-encoding plasmids or mRNA, or Cas9:gRNA complexed ribonucleoprotein (RNP), which allows for better control over the editing activity, shortening the window of editing time, which may potentially reduce off-target effects [45].

However, to promote HDR, a double or single stranded DNA donor template has to be transfected together with the CRISPR complex. For large DNA fragments, the Porteus group have shown efficient HDR levels in pluripotent stem cells [78,79], including iPSC [80], co-delivering CRISPR/Cas9 RNP and adeno-associated virus (AAV) donor templates. AAV6 seems to be the most efficient serotype to mediate homology template delivery in iPSC, yet AAV1, AAV2 and AAV-DJ can also promote high levels of transduction and should also be considered [80].

For base and prime editing, for which nucleoproteins are not currently commercially available, delivery of plasmids or chemically-modified mRNA:synthetic gRNA to iPSCs are both options. Plasmid nucleofection is an efficient technique that allows for the quick screening for editing efficiency but shows remarkable cell toxicity, while chemically modified mRNA:gRNA delivery has the advantage of generating viable cells with a better safety profile, due to the short time window in which the editing components are present in cells. Delivery of plasmid DNA or mRNA-encoding ABE or CBE was shown to precisely edit iPSC with over 90% efficacy and low indel rates (<1%), thus providing a rapid and more accessible manner to generate isogenic iPSC lines with minimal clone screening and low risk of off-target DNA breaks [81,82,83,84,85]. The delivery of prime editors in primary cells and iPSC works with variable efficacy [85,86,87,88]. Sürün and co-workers have shown that prime editors delivered as mRNA into iPSC were able to simultaneously edit two nucleotides to convert GFP to CFP with a modest 6–7.5% efficacy [85]. Chemello and colleagues were able to induce a +2-nucleotide insertion within the *DMD* gene with up to 54% efficacy following plasmid nucleofection [87]. Compared to base editors, prime editors seem to be less efficient, although no direct comparison has yet been performed in iPSC for the same target. This has been attributed to differences in repair mechanisms, which likely favor direct nucleotide transition over DNA recombination [75].

Overall, the fast-paced advances in CRISPR/Cas9-based editing methods have simplified the development of isogenic iPSC models of IRD to accurately investigate the role of known variants within the context of patient cells towards the discovery of novel therapeutics.

## 4. CRISPR-Mediated iPSC Editing for Inherited Retinal Disorders

One of the strengths of iPSC technology is the ability to model the effects of a variant in the native genetic background. However, even though iPSC systems offer a unique opportunity to study and annotate human genetic variants associated with disease, this also represents a major weakness. Experimental variability due to reprogramming or differentiation differences between lines reduce the biological significance of the comparison between these lines. In earlier iPSC studies, cell lines from unaffected family members or from age- or gender-matched unrelated controls, including either in-house or stem cell bank lines, were typically used as controls for patient-derived lines. However, transcriptional analyses using data from large stem cell repositories, such as the Human Induced Pluripotent Stem Cell Initiative (HipSci) or European Bank for Induced pluripotent Stem Cells (EBiSC), indicated that genetic differences between individuals have a greater influence in the variance than the differences observed between technical replicates, such as different clones from a single patient [89]. Even in cases where the cellular phenotype of a given mutation is strongly evident and highly penetrant, this may be lost due to genetic and epigenetic background differences [90,91,92,93]. In some cases, it is not possible to compare iPSC lines derived from patients with the disease and healthy controls, due to the inheritance patterns of linked single nucleotide polymorphisms (SNPs). Often, several SNPs are in linkage disequilibrium with each other, and they are always inherited together [94]. Besides the importance of isogenic iPSC to model a “disease-in-a-dish”, patients iPSC differentiated to retinal cells or 3D organoids can be used to test gene therapies and drugs for a potential treatment of the disease or for cell or sheet transplantation. The following sections will discuss the use of the CRISPR/Cas9 system in human iPSCs to target genes or genetic variants associated with IRDs, with a focus on Retinitis Pigmentosa (RP), Leber congenital amaurosis (LCA), X-linked retinoschisis (XLRS) and Usher Syndrome Type II (USH2), to model retinal development or contribute to the development of therapeutic approaches based on iPSC-derived retinal cells (Table 2).

### 4.1. CRISPR Technology to Generate a “Human Disease Model in a Dish”

The advent of CRISPR/Cas9 technology has improved the efficiency of genome editing and accelerated the generation of isogenic lines matched for origin, culture conditions, genetic background, epigenetic profile, differentiation capacity, etc. Gene-edited isogenic control lines differ from the patient iPSC at the site of genome editing only, and comparison between the two lines is ideal for the identification of disease mechanisms caused by a specific mutation. This has great relevance for the assessment of variant pathogenicity since it enables the comparison of two cell populations with identical genetic backgrounds and thus the appraisal of the impact of a specific single DNA lesion on the cellular phenotype [95]. Therefore, any differences observed between wild-type and mutant iPSCs can be more reliably attributed to the mutation itself, thus establishing a causal and more precise correlation between genotype and phenotype. Generating isogenic controls thus significantly reduces the variability that is introduced by differences in epigenetic and genetic background when using iPSCs from other sources. Furthermore, these lines are also extremely useful for transcriptomic analysis in order to identify novel disease-related pathways that can be targeted by future therapeutic strategies.

#### 4.1.1. CRISPR-Mediated Gene Knockout in Human iPSC

The knockout of a gene of interest is used to model loss-of-function variants and has enabled the interrogation of gene function in recessive disease (Figure 1E). Moreover, the use of gene-edited healthy iPSC can reduce costs and working time as obtaining patient samples and generating iPSCs are both costly and time consuming. This has been particularly helpful in rare diseases where there is reduced accessibility to patient-derived samples for the generation of patient-derived iPSCs or where there is difficulty in obtaining patient samples with a particular genotype, as well as for diseases in which the causative mutation disrupts proper reprogramming of patient-derived somatic cells to iPSCs. In addition, genome editing of control lines allows the study of many variants at once in the same genetic background, which may be more practical than collecting large numbers of patient lines [96].

CRISPR-mediated knockout of a gene of interest was pursued to investigate the function of the *KCNJ13* gene in relation to RPE structure and phagocytic activity as an RPE model of Leber congenital amaurosis type 16 (LCA16). Kanzaki and colleagues [29] used CRISPR/Cas9 gene editing in healthy iPSCs to knock out *KCNJ13* and observed a decrease in the phagocytic activity and expression of phagocytosis-related genes recapitulating the LCA16 phenotype. The same genome editing strategy was also employed to determine the effect of lack of connexin 43 (CX43) on retinal development [97]. Mutations in the CX43-coding gene, *GJA1*, have been associated with oculodentodigital dysplasia (ODDD), including microphthalmia and other ocular defects such as iris atrophy, glaucoma, strabismus and blindness. To delineate the role of CX43 in retinal development, the authors disrupted *GJA1* in healthy donor iPSCs by targeting *GJA1* exon 2 with CRISPR/Cas9, delivered by plasmid nucleofection, and obtained approximately 86% (6 out of 7 clones) of homozygous gene targeting. Although the expression of pluripotency markers in *GJA1*−/− iPSCs was comparable to control iPSCs, the ROs differentiated from the ablated line were smaller in size, with a decreased abundance of all mature retinal cell types.

#### 4.1.2. CRISPR/Cas9 HDR to Generate Isogenic Corrected Human iPSC as Controls

Gene correction in patient-derived iPSC has the advantage that for recessive or dominant disease, only one allele needs to be repaired. CRISPR technology has been extensively used to genetically correct mutations in iPSC derived from patients affected by retinal disorders (Figure 1F).

Four years after the first applications of the CRISPR/Cas9 system, Bassuk et al. [98] used this genome editing tool to precisely repair, in patient-specific iPSCs, a nonsense point mutation in the retinitis pigmentosa GTPase regulator (*RPGR*) gene that causes the X-linked variant of Retinitis Pigmentosa (XLRP). The Tsang group exploited SpCas9/gRNA and a single stranded oligonucleotide (ssODN) template carrying the correct nucleotide sequence to trigger homology direct repair (HDR). Although the efficiency of the gene correction was quite low (13%), this study supported the development of personalized iPSC-based transplantation therapies for retinal disease. A step forward, which followed the correction by CRISPR and HDR of two mutations in the *RPGR* gene, was reported by Deng WL and colleagues [99]. The authors differentiated gene-corrected patient urinary iPSCs to RPE cells and generated 3D ROs rescuing photoreceptor structure and electrophysiological properties, reversing the observed ciliopathy and restoring gene expression to a level in accordance with that in the control using transcriptome-based analysis.

A study by Foltz and colleagues [100] describes the use of genome editing technology in patient-derived iPSCs to correct the c.6901C>T point mutation in the *PRPF8* gene that causes Retinitis Pigmentosa 13 (RP13), thus generating an isogenic control line for cellular modelling. Interestingly, the strategy for the correction included electroporation of iPSCs with a plasmid carrying a PRPF8-specific gRNA that overlaps the patient-specific mutation, mRNA-encoding Cas9-Gem to facilitate low and transient expression of the nuclease and an ssODN repair template. Cas9-Gem is a SpCas9 variant engineered to be degraded in the G1 phase and thus to decrease the frequency of events repaired by NHEJ [101]. The authors successfully generated RPE from patient and corrected iPSCs (derived from fibroblasts) and observed similar differentiation, morphology and apicobasal polarity in both lines. RPE cells from diseased and corrected iPSCs did not show different functionality in terms of phagocytosis of fluorescently labelled photoreceptor outer segments, and secretion of PEDF and MGF-E8.

The comparison between patient-derived iPSCs, healthy donors and CRISPR-corrected controls was instrumental to delineate the molecular and cellular mechanisms underlying the pathology of *PRPF31* splicing factor-related Retinitis Pigmentosa 11 (RP11). First, Buskin and colleagues [102] corrected the c.1115_1125del11 mutation in the *PRPF31* gene that causes RP11 using a CRISPR/HDR strategy. The authors, using ssODN, reintroduced the wild-type *PRPF31* sequence and reprogrammed patient fibroblasts into iPSCs, and patient and corrected iPSCs were differentiated into ROs and RPE. They observed that HDR-based CRISPR-correction resulted in the rescue of molecular and cellular phenotypes. The same iPSC lines were further characterized by Georgiou and colleagues, who showed localization of mutant PRPF31 in cytoplasmic aggregates in RP11 iPSC-RPE cells, in contrast to control cells where PRPF31 was localized in nuclear speckles required for splicing activity [103]. The authors also demonstrated that the dysregulation of splicing promoted protein misfolding and contributed to aggregate formation. Interestingly, the activation of the autophagy pathway using Rapamycin resulted in the reduction of cytoplasmic aggregates and improvement of cell survival.

Isogenic iPSCs lines with heterozygous and homozygous correction of c.992_993delCA mutation in the *MERTK* gene causing autosomal recessive retinitis pigmentosa (arRP) were generated by Artero-Castro A. and colleagues [104]. These genetically corrected iPSCs represent accurate controls to study the contribution of the specific genetic change to the disease. The authors nucleofected patient iPSCs bearing the c.992_993delCA mutation in the *MERTK* gene with an RNP complex consisting of the eSpCas9 protein, chemically synthesized crRNA and tracrRNAs and an ssODN carrying the correct sequence. The heterozygous and homozygous gene-corrected isogenic iPSC lines displayed a typical human embryonic stem cell (hESC) colony-like morphology, a normal karyotype and could be differentiated into the three germ layers (mesoderm, ectoderm and endoderm) in vitro. More recently, Artero-Castro A et al. [105] differentiated a gene-corrected iPSC line into RPE cells and showed that the homozygous correction led to the recovery of the expression of MERTK protein and phagocyte function to levels comparable to wild-type iPSC-RPE.

To model X-linked retinoschisis (XLRS), Huang and co-workers [106] took advantage of the CRISPR/Cas9 system to generate iPSC lines from peripheral blood mononuclear cells (PBMCs) isolated from two patients carrying mutations in *RS1* at c.625C>T (p.R209C) and c.488G>A (p.W163X), respectively, and further established 3D ROs. To confirm the genotype–phenotype relationship, they generated isogenic controls by correcting the *RS1* c.625C>T mutation in patient-specific iPSCs with plasmid-based delivery of the CRISPR/Cas9 system together with HDR templates. The strategy proved efficient, with more than 50% of transfected cells showing the repaired genotype, but indels were detected in many uncorrected clones. For this reason, the authors tested base editors for repairing the *RS1* mutation, which showed over 50% efficiency without detection of indels in the target genomic region. The generation of an isogenic control was instrumental for the cellular modelling of XLRS as *RS1*-repaired clones displayed reversion of all the molecular and structural deficiencies observed in patient-derived ROs.

A similar approach based on HDR triggered by CRISPR/Cas was shown in patient-derived iPSCs affected by a mutation causing arRP. To correct a homozygous Alu insertion in exon 9 of the male germ cell-associated kinase (*MAK*) gene, patient iPSCs were transfected with a plasmid expressing SpCas9, gRNA and an HDR donor plasmid. Following puromycin selection, 16% of colonies showed monoallelic correction resulting in the restoration of exon 9-containing transcript and full-length MAK protein [107].

A CRISPR/HDR-mediated strategy was also pursued to correct two highly frequent mutations (c.119-2A>C splice site mutation and the p.Arg75Ser variant) in the *NR2E3* gene, a transcription factor essential for photoreceptor development and maintenance, causing Enhanced S Cone syndrome (ESCS). iPSC lines generated from four patients were corrected at the genomic level, restoring the expression of the WT *NR2E3* transcript from one allele [108].

More recently, the two most prevalent *USH2A* mutations accounting for half of USH2 cases were corrected by the CRISPR/HDR approach in patients’ iPSCs as proof of concept for future autologous cell therapy [109]. The authors used eSpCas9 and ssODN to correct c.2276G>T (p.Cys759Phe) and c.2299delG (p.Glu767Serfs*21) located 22 bp apart in exon 13. Upon nucleofection, EGFP-positive iPSCs were cell-sorted and cloned. The few surviving clones showed scarless HDR but interestingly, the corrected lines retained normal genomic stability and pluripotency. The same approach was described for the correction of the *USH2A* c.2299delG mutation in USH2 iPSCs established from PBMC of a patient carrying compound heterozygous *USH2A* c.2299delG and c.1256G>T variants. HDR-mediated correction was achieved by transfection of SpCas9 RNP together with ssODN and showed 15% efficiency of correction of the mutation [110].

A similar HDR-based CRISPR/Cas9 strategy was used in order to homozygously correct the biallelic c.834G>A, p.Trp278X mutation with an efficiency of 30% in the *AIPL1* gene that causes Leber Congenital Amaurosis type 4 (LCA4) [111]. The authors reprogrammed renal epithelial cells into iPSCs and differentiated them into ROs. They observed restoration of AIPL1 protein expression in this isogenic repaired line and used it as valuable control to test the translational readthrough-inducing drug PTC124 as a potential therapy for LCA4.

#### 4.1.3. CRISPR/Cas9-Mediated NHEJ to Target Mutated Allele in Patient-Derived iPSC

A different strategy based on the repair of a mutation via CRISPR-mediated NHEJ was proposed for the most common loss-of-function mutation in the *CEP290* gene causing the autosomal recessive retinal disorder LCA10 [107]. Patient-derived iPSCs homozygous for the c.2991 + 1655A>G mutation in *CEP290* (IVS26), located within intron 26 and resulting in the inclusion of a cryptic exon, were treated with two gRNAs and SpCas9 targeting sites flanking the IVS26 mutation to remove the mutated nucleotide by CRISPR-mediated indel formation. More than 50% of the clones isolated from treated iPSCs showed loss of the disease-causing mutation in at least one of the *CEP290* alleles and restoration of splicing and protein expression. Interestingly, the authors proposed the same strategy employing the *Staphylococcus aureus* Cas9 (SaCas9) compatible with in vivo delivery mediated by AAV and confirmed the removal of the IVS26 mutation in patient-derived iPSCs.

A number of recent studies have also investigated CRISPR-mediated NHEJ repair to treat autosomal dominant genetic mutations in patients’ iPSCs. The c.166G>A (p.G56R) dominant mutation in the *NR2E3* gene, accounting for all NR2E3-associated adRP cases and representing the second most common mutation causing adRP, was corrected in patient-derived iPSC using the CRISPR/Cas system. The authors transfected a plasmid expressing the mutation-specific gRNA, SpCas9 and EGFP in patient-derived iPSC. Upon EGFP sorting and cloning, four colonies were obtained, and two of them (50%) carried the allele-specific knockout. The G56R-CRISPR iPSC lines retained genetic stability and pluripotency and were successfully differentiated to NR2E3-expressing ROs without off-target effects due to editing [112]. CRISPR-triggered NHEJ was also successfully employed in patient iPSC to eliminate the cone-rod homeobox (*CRX*) allele carrying the dominant c.262A>C (p.K88Q) mutation causing LCA7 and accounting for 2% of all LCA cases [113]. The authors introduced two DSBs, one specific for the c.262A>C (p.K88Q) mutation and one for an SNP present in the mutated allele, to knockout the expression of the mutant allele and preserve the expression of the wild-type protein. The CRX+/− and control CRXK88Q/+ iPSCs were differentiated to ROs. Although the authors observed a mild delay in photoreceptor maturation, they demonstrated a moderate rescue of photoreceptor cell development and maturation in ROs.

#### 4.1.4. CRISPR Technology to Generate Mutated Human iPSC

Insertion of an “on-target” mutation in healthy iPSC represents a valuable strategy to demonstrate the disease-causing effect of the mutation and to study the molecular/cellular pathways that lead to the disease (Figure 1G). The causative role of *RS1* mutations for XLRS was unraveled by Huang, K.C et al., who introduced the *RS1* c.625C>T mutation into a healthy donor iPSC line with CRISPR/Cas9 and generated ROs which fully exhibited the XLRS phenotype [106]. Similarly, Yang and colleagues introduced the *RS1* c.625C>T mutation in healthy iPSCs but they employed nanodiamonds as the delivery system for the CRISPR/Cas9 components and HDR template. The authors reported an editing efficiency of more than 19% without affecting iPSC viability and concluded that this can be successfully used as a tool for creating in vitro and in vivo disease models of XLRS [114].

To test the use of AAV-mediated gene augmentation therapy for the treatment of *PRPF31*-associated retinal degeneration, Brydon and colleagues knocked out the *PRPF31* gene using CRISPR/Cas9 genome editing in healthy iPSC. The authors selected a heterozygous 10bp deletion allele in exon 7 generating a premature stop codon in exon 8, differentiated these cells into RPE and showed restoration of functionality in cells treated with AAV-PRPF31 [115].

Similarly, to test an AAV-based gene addition strategy for XLRP caused by nonsense mutation c.358C>T (p.R120X) in the *RP2* gene, Lane and co-workers generated isogenic RP2 knockout (RP2 KO) patient-derived iPSCs [116]. The gene addition strategy performed in animal models for RP2 XLRP using an AAV RP2 vector demonstrated the preservation of cone function but had no effect on rods, indicating that the available animal models for RP2 XLRP do not recapitulate the severe phenotype observed in some patients experiencing macular atrophy in childhood. The authors thereby generated gene-edited isogenic RP2 KO iPSCs by introducing a DSB in exon 2 of the *RP2* gene. RP2 KO iPSCs and, as a control, RP2 patient-derived iPSC, were used to produce 3D ROs as a human retinal disease model. Interestingly, RP2 ablation did not prevent the differentiation of iPSC into photoreceptors bearing outer segment-like structures in 3D RO culture, even if the loss of RP2 induced cell death in ROs primarily affecting rods and not cones. Upon a deep characterization of RP2 KO and patient-derived iPSC, the authors used these cells as an in vitro model to test AAV-mediated augmentation of RP2 expression before the onset of outer nuclear layer (ONL) thinning. Forty days later, the ONL thickness was similar to that observed for the isogenic control cells, suggesting near-complete rescue.

### 4.2. CRISPR Technology to Model Retinal Development

CRISPR-mediated HDR has also been exploited to generate iPSC lines expressing reporter genes in specific retinal cell populations to dynamically study the development of ROs. The PGP iPSC line is a triple transgenic reporter line where the stop codons of visual system homeobox 2 (*VSX2*) (marking neural retinal progenitors), POU class 4 homeobox 2 (*POU4F2*/*BRN3b*) (specific for retinal ganglion cells) and recoverin (*RCVRN*) (expressed by photoreceptors) were replaced with sequences encoding the P2A peptide fused to Cerulean, green fluorescent protein (GFP) and mCherry reporter genes, respectively [117]. ROs from the PGP iPSC line were monitored across time, and the dynamics of the expression of the reporters facilitated visual observation of retinal differentiation. Thus, the PGP line represents a tool for studying retinal development, disease modelling and therapeutic drug screening. Another dual reporter iPSC line was generated to express GFP and tdTomato sequences coupled to SIX6 and POU4F2, respectively [118]. The SIX6-GFP labelled the whole developing retina and allowed the optimization of microenvironment conditions (including hypoxia, Wnt and SHH signalling pathways) for proper optic vesicle development. The SIX6-GFP/POU4F2-tdTomato dual reporter line was instrumental in confirming the retinal development of SIX6-GFP cells, distinguishing them from non-retinal organoids.

### 4.3. Gene-Edited iPSC Differentiated into Retinal Cells for Transplantation

iPSC-derived RPE cell sheets have already been tested in a safety and tolerability perspective clinical trial to evaluate their subretinal transplantation in AMD patients (NCT0433976). However, iPSCs derived from patients affected by IRDs carry pathogenic variants that need genetic correction before differentiation and transplantation. CRISPR/Cas9 technology could serve as a tool to edit and correct causative genetic variations in patient-derived iPSCs to provide personalized therapy for the replacement of precursor cells in a degenerating retina, an application not described in the pre-clinical setting so far. Meanwhile, the CRISPR/Cas9 system has been employed to improve the identification and transplantation of iPSC-derived retinal cells. Matsuyama and colleagues pursued CRISPR-mediated knockout of *Bhlhb4* and *Islet1* genes (*Bhlhb4*−/− and *Islet1*−/−), crucial for neural maturation of rod and cone bipolar cells, in pluripotent cell lines (both iPS and ES cells) to improve the outcome of ESC/iPSC-retinal sheet transplantation in restoring visual function in mice with end-stage retinal degeneration [119]. To prepare retinal sheets with fewer bipolar cells that directly receive the output from the photoreceptor cells, the authors knocked out *Bhlhb4* and *Islet1* genes (*Bhlhb4*−/− and *Islet1*−/−). They designed gRNAs for each gene and nucleofected the CRISPR/gRNA components to iPS and ES cells. Upon antibiotic selection, they obtained at least two or three clones for each line bearing a biallelic knockout of the desired genes. The retinal sheets were transplanted in end-stage *rd1* mouse retinas in order to improve the first synaptic transmission in the reconstructed retina from graft photoreceptors to host bipolar cells. Overall, the authors demonstrated that CRISPR KO cell lines can be used to improve neural integration of retinal sheet transplantation.

With a view to develop a cell therapy product to treat retinal disorders with dysfunction or loss of photoreceptors, CRISPR/Cas9 has been coupled with iPSC technology to study RO development and identify cell surface markers of precursors or mature photoreceptors. Gagliardi and colleagues generated an iPSC line from adult Muller glial cells engineered with CRISPR/Cas9 to express nuclear mCherry-H2B under the control of the *CRX* promoter inserted in both AAVS1 loci [120]. The AAVS1:CrxP H2BmCherry hiPSC line allowed the differentiation of photoreceptor precursors to retinal ganglions to be followed, confirming that CD73 expression is restricted to cells expressing CRX and thus to photoreceptor precursors and differentiated cones and rods. The authors transplanted CD73+ cells in a rat model of retinal degeneration bearing the P23H-mutated *Rho* transgene and showed the survival and maturation of a homogenous population of RCVRN-positive human cells highly enriched in red/green cones. The authors proposed CD73 as a marker which could be employed to sort photoreceptor precursors that could be safely transplanted in the host retina.

To the same end, Guan and colleagues [121] used CRISPR/Cas9 to engineer an iPSC line expressing a photoreceptor-specific reporter by targeting exon 3 of the locus coding for the pan photoreceptor marker RCVRN and inserting a P2A-EGFP sequence. The clone selected by puromycin resistance showed a normal karyotype, expression of typical pluripotency markers and multilineage differentiation ability, as well as induction of ROs, which was dynamically monitored to follow and identify RCVRN-EGFP+ photoreceptor precursors. Transcriptomic analysis of sorted EGFP+ and EGFP− cells allowed the identification, as well as the exclusion, of photoreceptor biomarkers. The authors indeed reported that CD73 was expressed by both sorted populations, while suggesting that CD133 would represent a favorable biomarker for the enrichment of human photoreceptors. Although the authors did not transplant CD133+ cells, the identification of a new biomarker would contribute to the development of stem cell-based therapy to replace photoreceptors in the retina.

**Table 2:** Applications of CRISPR/Cas9 technologies combined with iPSCs in the study and treatment of retinal degenerative diseases.

Publication	iPSC Source	Retinal Disease	Gene; Mutation; Patient Information	CRISPR-Mediated Genome Editing: Mechanism and Delivery
Bassuk et al., 2016 [98]	Dermal skin fibroblasts	XLRP	*RPGR* c.3070G>T	HDR correction of mutation. Transfection with plasmid for SpCas9 and gRNA expression and ssODN template.
Burnight et al., 2017 [107]	Dermal skin fibroblasts	RP, LCA10	353-bp Alu insertion in *MAK* (Patient 1) *CEP290* c.2991 + 1655 A>G, IVS26 (Patient 2–4) *RHO* c.163 C>A (Patient 5)	HDR correction of mutation 353-bp Alu insertion in *MAK*, by nucleofection with plasmids for SpCas9 and gRNA expression and HDR template. NHEJ-mediated repair and HDR correction of IVS26 mutation, by transfection of plasmids for SpCas9 or SaCas9 and single or dual gRNA expression. Knockout and HDR correction of *RHO* c.163 C>A.
Buskin et al., 2018 [102]	Dermal skin fibroblasts	RP11	*PRPF31* c.1115_1125 del11 (Patient 1–3) *PRPF31* c.522_527+10del (Patient 4)	HDR correction of c.1115_1125 del11 mutation in a patient-specific iPSC line. Transfection of plasmid for SpCas9 and gRNA expression and ssODN template.
Deng et al., 2018 [99]	Renal epithelial cells	XLRP	*RPGR* c.1685_1686delAT (Patient 1) *RPGR* c.2234_2235delGA (Patient 2) *RPGR* c.2403_2404delAG (Patient 3)	HDR correction of c.1685_1686delAT mutation. Nucleofection with plasmids for SpCas9 and gRNA expression and HDR template.
Foltz et al., 2018 [100]	Dermal skin fibroblasts	RP13	*PRPF8* c.6901C>T	HDR correction of mutation. Electroporation with plasmids for gRNA expression, Cas9-Gem mRNA and ssODN template.
Gagliardi et al., 2018 [120]	Retinal Müller glial cells	/	*AAVS1*	CRISPR-mediated HDR to introduce a reporter gene under the control of murine *Crx* promoter, in AAVS1 site. Transfection with plasmids for SpCas9 and gRNA expression and HDR template.
Artero-Castro et al., 2019 [104]	Dermal skin fibroblasts	arRP	*MERTK* c.992_993delCA	HDR correction of mutation. Transfection with eSpCas9_1.1 RNP and ssODN template. Clones carrying homozygous and heterozygous correction were obtained.
Bohrer et al., 2019 [108]	Dermal skin fibroblasts	ESCS	*NR2E3* c.119-2A>C (Patient 1) *NR2E3* c.219G>C and c.932G>A (Patient 2)	HDR correction of c.119-2A>C (Patient 1) and c.219G>C (Patient 2) mutations. Transfection with plasmids for SpCas9 and gRNA expression and HDR template.
Brydon et al., 2019 [115]	Dermal skin fibroblasts	RP11	*PRPF31* exon 7 (Healthy donor) *PRPF31* c.1115_1125del11(Patient)	Knockout of *PRPF31* by targeting exon 7 in wt iPSC to create a haploinsufficient *PRPF31*+/- line, by nucleofection with plasmids for SpCas9 and gRNA expression. RP11-iPSC line carrying c.1115_1125 del11 mutation and corrected by CRISPR-mediated HDR (Buskin et al., 2018).
Huang et al., 2019 [106]	PBMC	XLRS	*RS1* c.625C>T (Patient 1) *RS1* c.488G>A (Patient 2)	Correction of c.625C>T mutation in patient iPSC by CRISPR-mediated HDR and also an adenine base editing approach. Introduction of c.625C>T mutation in wt iPSC. Nucleofection of plasmids for Cas9, gRNA and HDR templates and ABE7.10 base editor.
Kanzaki et al., 2020 [29]	Dental pulp cells	LCA16	*KCNJ13*	Knockout of *KCNJ13* by targeting exon 2 and 3 in wt iPSC. Electroporation with SpCas9 protein and gRNA expression.
Lam et al., 2020 [117]	Dermal skin fibroblasts	/	*VSX2* *BRN3b* *RCVRN*	CRISPR-mediated HDR to introduce reporter genes in targeted loci. Transfection with plasmids for SpCas9 and gRNA expression and HDR templates.
Lane et al., 2020 [116]	Dermal skin fibroblasts	XLRP	*RP2* exon 2 *RP2* c.358C>T	Knockout of *RP2* by targeting exon 2 in wt iPSC. Electroporation with plasmid for SpCas9 and gRNA expression.
Sanjurjo-Soriano et al., 2020 [109]	Dermal skin fibroblasts	USH2A	*USH2A* c.2299delG (Patient 1) *USH2A* c.2276G>T and c.2299delG (Patient 2)	HDR correction of *USH2A* mutations. Nucleofection with plasmid for eSpCas9(1.1) and ssODN template.
Yang et al., 2020 [114]	PBMC	XLRS	*RS1*	Introduction of RS1 c.625C>T in wt iPSC. Nanodiamond carriers of linear DNA for CRISPR components and HDR template.
Artero-Castro et al., 2021 [105]	Dermal skin fibroblasts	arRP	*MERTK* c.992_993delCA	iPSC lines carrying c.992_993delCA mutation and homozygously or heterozygously corrected by CRISPR-mediated HDR (Artero-Castro et al., 2019).
Chirco et al., 2021 [113]	PBMC	LCA7	*CRX* c.464_465insGGCA *CRX* c.262A>C	Knockout of *CRX* c.262A>C mutated allele. Transfection with plasmids for SpCas9 and gRNA expression.
Diakatou et al., 2021 [112]	Dermal skin fibroblasts	adRP	*NR2E3* c.166G>A	Knockout of *NR2E3* mutated allele. Transfection with plasmid for SpCas9 and mutation-specific gRNA expression.
Liu et al., 2021 [110]	PBMC	USH2A	*USH2A* c.2299delG and c.1256G>T	HDR correction of *USH2A* c.2299delG mutation. Transfection with SpCas9 RNP and ssODN template.
Matsuyama et al., 2021 [119]	Murine fibroblasts	/	*Bhlhb4* *Islet1*	Knockout of *Bhlhb4* and *Islet1* in Tg(*Nrl*-GFP); Ribeye-reporter miPS cells and *Thy*1-GCaMP6f; Ribeye-reporter mES cells. Nucleofection with plasmids for SpCas9 and gRNA expression.
Wahlin et al, 2021 [118]	Fibroblasts	/	*SIX6* *POU4F2*	CRISPR-mediated HDR to introduce reporter genes in targeted loci. Transfection with plasmids for SpCas9 and gRNA expression and HDR templates.
Cheng et al., 2022 [97]	Dermal skin fibroblasts	ODDD	*CX43*	Knockout of *CX43* in wt iPSC. Nucleofection with plasmid for SpCas9 protein and gRNA expression.
Guan et al., 2022 [121]	Bone marrow CD34+ cells	/	*RCVRN*	CRISPR-mediated HDR to introduce a reporter gene in the targeted locus. Transfection with plasmids for SpCas9 and gRNA expression and HDR template.
Georgiou et al., 2022 [103]	Dermal skin fibroblasts	RP11	*PRPF31* c.1115_1125 del11 (Patient 1–3) c.522_527+10del (Patient 4)	RP11-iPSC line carrying c.1115_1125 del11 mutation and corrected by CRISPR-mediated HDR (Buskin et al., 2018).
Leung et al., 2022 [111]	Renal epithelial cells	LCA4	*AIPL1* c.834G>A (Patients 1) *AIPL1* c.834G>A and c.466-1G>C (Patients 2–3) AIPL1 c.834G>A and c.665G>A (Patients 4)	HDR correction of mutation to create isogenic control lines. Nucleofection with eSpCas9_1.1 RNP and ssODN template.

## 5. Concluding Remarks

The studies here reviewed the current state of the CRISPR/Cas9 system as a useful and powerful tool for editing the genome of iPSC. Several sources of healthy and patient cells for iPSC reprogramming are available to date. Fibroblasts remain the most commonly used source but renal epithelial cells and dental pulp are also easily accessible tissues to be reprogrammed to iPSC. In the last 5–6 years, the CRISPR system has evolved rapidly and extensively. Cas nucleases belonging to different types of the Class 2 effector complex have been isolated, characterized and engineered for applications in eukaryotic cells and in animal models. Moreover, high-fidelity variants of SpCas9 have been generated to expand its recognition to non-NGG PAM sequence. The development of base editing and the double-programmable prime editing have revolutionized CRISPR applications, breaking down the limitations of the first era of CRISPR.

The combination of the CRISPR system with iPSCs has promising applications in gene and cell therapy for retinal degeneration diseases. Isogenic iPSCs can faithfully model a “disease-in-a-dish” demonstrating the mutation-causative effect. The generation of isogenic pairs of genome-edited iPSC lines has been used successfully for investigating disease mechanisms in IRDs, for modelling retinal development and for testing drug and gene therapies. Moreover, gene-corrected patient iPSCs differentiated to retinal cells (e.g., RPE and photoreceptors) provide a promising cell transplantation treatment option for retinal diseases.

## Figures and Tables

**Table 1:** Sources of healthy donor or patient cells for iPSC reprogramming.

Publication	iPSC Source	Reprogramming Method	Donor Info; Gene; Mutation
Yoshida et al., 2014 [28]	Dermal skin fibroblasts	Retroviral transduction	RP patient, *RHO* c.541G>A
Geng et al., 2017 [27]	Conjunctival cells	Sendai virus	AMD patients and healthy donor
Capowski et al., 2019 [23]	Blood/Fibroblasts	Episomal vectors	RP/LCA/Usher syndrome patients and healthy donors
Slembrouck-Brec et al., 2019 [30]	Post-mortem adult Müller glia	Sendai virus	Healthy donor
Kanzaki et al., 2020 [29]	Dental pulp cells	Episomal vectors	Healthy donor

## Data Availability

Not applicable.
